# Case report of squamous cell carcinoma secondary to recurrent anal fistula

**DOI:** 10.3389/fonc.2025.1673829

**Published:** 2025-10-02

**Authors:** Qin-Bing Zhu, Jing Zhang, Chen-Yuan Liu, Hui Ye, Shi-Ping Huang, Ya-Hong Xue

**Affiliations:** ^1^ Graduate School, Nanjing University of Chinese Medicine, Nanjing, Jiangsu, China; ^2^ Anorectal, Nanjing Hospital of Chinese Medicine Affiliated to Nanjing University of Chinese Medicine, Nanjing, Jiangsu, China

**Keywords:** anal fistula, malignant transformation, squamous cell carcinoma, case, secondary

## Abstract

Anal fistula is a common benign perianal condition, and its malignant transformation, particularly into squamous cell carcinoma, is rare and often associated with comorbidities such as Crohn’s disease and HIV infection. This article reports the case of a 66-year-old male with a history of recurrent anal fistula spanning more than 10 years who underwent 3 surgical treatments. Pathological findings from the first two surgeries showed no malignant lesions, while the third confirmed moderately to poorly differentiated squamous cell carcinoma. The patient had a history of lymphoma and small bowel resection; HIV infection was excluded upon admission. Carcinoembryonic antigen and cytokeratin 19 fragment levels were mildly elevated, and imaging revealed a high complex anal fistula. The diagnosis was ultimately confirmed by pathological biopsy. We propose that malignant transformation may be linked to chronic inflammatory stimulation, reduced immune function, and prior abdominal surgery. Diagnosis depends on pathological confirmation, and the use of multiple imaging modalities can improve diagnostic accuracy. The uniqueness of this case lies in the non-continuous recurrence of the anal fistula, offering a new perspective on the clinical characteristics of anal fistula carcinogenesis.

## Introduction

Anal fistula is a common perianal condition with a generally favorable prognosis; however, malignant transformation can occur. The overall incidence of anal fistula-related cancers is reported to be 0.3%–0.7%. Risk factors for malignant transformation include colorectal disease, chronic perianal conditions, and human papillomavirus (HPV) infection (1, 2). The two most common malignancies arising from anal fistula are adenocarcinoma and squamous cell carcinoma. According to previous literature reviews and case reports, squamous cell carcinoma associated with anal fistula is extremely rare (3, 4). Most reported cases are complicated by Crohn’s disease, HIV infection, and other immunosuppressive conditions (2, 5). Only two cases of squamous cell carcinoma arising from a simple anal fistula have been documented. One, reported by Seya et al. in 2007, involved a patient with a 32-year history of perianal abscess who died of bladder cancer 10 years after anal fistula resection (6). The second, reported by Chen-guo Zheng et al., described a patient with a 20-year history of recurrent perianal discharge who was treated with Mohs micrographic surgery, although no postoperative outcomes were reported (7). This article presents a case of anal fistula-associated squamous cell carcinoma in a patient who underwent three fistula surgeries over a decade. Pathological examinations from the first two surgeries showed no specific lesions, while the final surgery revealed squamous cell carcinoma.

## Case presentation

In May 2025, a 66-year-old male patient was admitted to the hospital with an anal fistula. His medical history included hypertension and diabetes mellitus. In 2020, he had undergone small bowel resection for –lymphoma. Over a decade earlier, he had anal fistula surgery at an external hospital, where postoperative pathology revealed no abnormalities, and the wound healed well. In 2023, he presented with perianal swelling and pain and was diagnosed with an anal fistula. In 2024, he underwent another surgical procedure at the same hospital; the postoperative wound healed well, and pathology revealed no specific changes. One month prior, the patient developed a mass at the original surgical site, accompanied by pain, discomfort, and minimal fluid discharge. Bowel movements occurred every other day, one to two times each day, with occasional bright red hematochezia. On physical examination, the anal region appeared uneven, with an old surgical scar visible at the 3 o’clock position in the lithotomy position. A firm, irregularly surfaced mass measuring approximately 1 × 1 cm was located 2 cm from the anal margin at the 3 o’clock position, with local redness, swelling, and minimal discharge. Digital rectal examination revealed a mass at the same position, with slight blood staining on the glove upon withdrawal. No other abnormalities were detected.

The admission diagnosis was suspected to be an anal fistula. Laboratory tests revealed a carcinoembryonic antigen level of 9.19 ng/mL and a cytokeratin (CK) 19 fragment level of 5.36 ng/mL. Infectious disease screening excluded HIV infection. Two-dimensional endorectal ultrasound showed a subcutaneous hypoechoic area at the 3 o’clock position in lithotomy, extending through the sphincter and submucosa to the anorectal ring from the 3 to 5 o’clock positions, with local blood flow signals—findings consistent with a high complex anal fistula. Pathological examination was recommended to rule out malignancy. To further clarify the diagnosis, colonoscopy was performed. A sessile polyp approximately 0.8 × 0.8 cm in size with a smooth surface was found in the transverse colon near the hepatic flexure. Additionally, a mucosal ulcer was observed in the transverse colon 70 cm from the anus, and a biopsy was taken. In the sigmoid colon, 18 cm from the anus, a pedunculated polyp measuring approximately 1.2×1.0 cm with a smooth, congested, and lobulated surface was identified. No other abnormalities were detected. The colonoscopy findings and clinical presentation excluded Crohn’s disease. Post-colonoscopy pathology revealed a tubular adenoma in the transverse colon, with areas of low-grade intraepithelial neoplasia in the glandular epithelium.

Pelvic contrast-enhanced magnetic resonance imaging (MRI) revealed perianal and gluteal soft tissue swelling. An internal opening was observed at the 3–7 o’clock position of the anal canal at the dentate line level, extending superiorly through the sphincter to the inferior levator ani and inferiorly to the subcutaneous layer of the intergluteal groove. The lesion demonstrated diffusion restriction, with its widest portion (approximately 10 mm in diameter) located in the intersphincteric space between the internal and external anal sphincters, and it showed marked enhancement following contrast administration. Although bladder distension was suboptimal, the bladder wall appeared smooth with no abnormal signal intensity within the lumen. A focal area of decreased T2 signal was noted in the peripheral zone of the prostate. The seminal vesicles showed no obvious abnormalities. A small amount of pelvic effusion was also present. During surgery, as a pathological biopsy of the anal mass had not been performed under colonoscopy, a high complex anal fistulotomy with seton placement was carried out under lumbar anesthesia. Intraoperative exploration revealed that the cavity extended directly to the mass located above the dentate line at the 3 o’clock position. The firmer tissue in this area was resected and submitted for pathological analysis. The fistula tract was fully opened along its course to the vicinity of the 3 o’clock anal sinus using electrocautery, and tract wall tissue was excised and sent for pathological examination.

**Figure 1:**
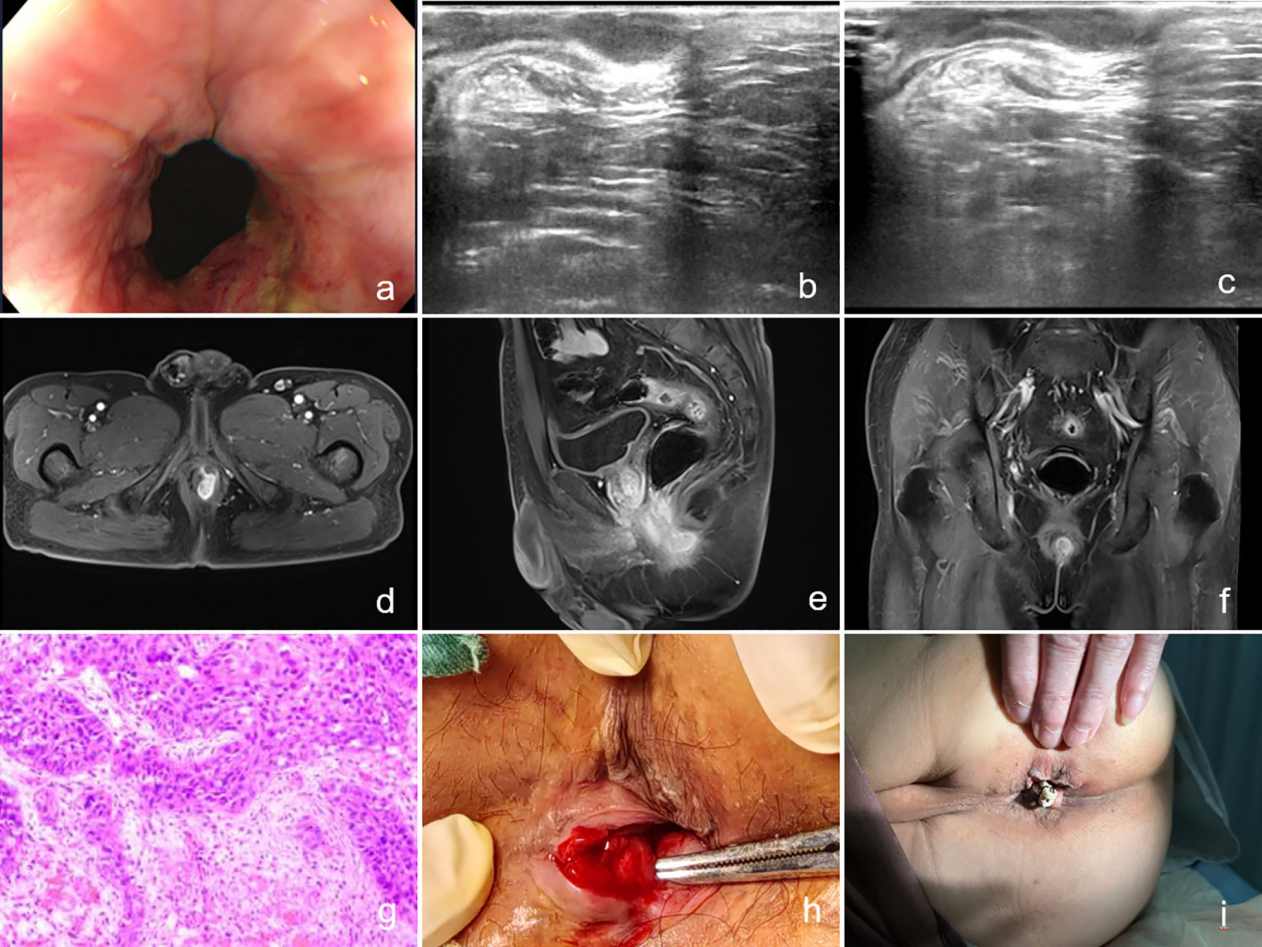
**(a)** Image of the anal orifice taken by colonoscopy; **(b, c)** Images from two-dimensional endorectal ultrasound; **(d–f)** Magnetic resonance images; **(g)** Postoperative pathology; **(h)** Image on the day of surgery; **(i)** Image on the second postoperative day.

Due to significant involvement of the sphincter muscles, a rubber band seton was placed at the internal opening above the dentate line at the 3 o’clock position. Postoperative pathology revealed the following: (1) Biopsy tissue from the anal fistula demonstrated moderately to poorly differentiated squamous cell carcinoma; (2) Biopsy tissue from the anal mass also showed moderately to poorly differentiated squamous cell carcinoma. Immunohistochemistry results were as follows: CK (3+), CK5/6 (3+), P40 (3+), CK20 (−), CK7 (2+), S-100 (−), Melan-A (−), and Ki-67 (60%+). The patient was advised to seek further treatment at a specialized center for comprehensive management, including radiotherapy and chemotherapy. The imaging data, pathological section images, and intraoperative as well as postoperative photos related to the patient's condition are detailed in **Figure 1**.

## Discussion

The mechanism underlying anal fistula carcinogenesis remains unclear. In 1974, Rosser first reported six cases and proposed diagnostic criteria: (1) persistent presence of the fistula; (2) absence of tumors on the luminal surface of the rectal canal; and (3) absence of malignant tissue in the internal opening of the fistula tract (8). It is currently believed that anal glands serve as the tissue origin for carcinogenesis in anal fistulas (9). Recurrent inflammatory stimulation and abnormal scar tissue changes are considered key contributing factors. In an environment rich in inflammatory cells, sustained cellular proliferation significantly increases the risk of tumorigenesis. A decline in immune function is also thought to contribute to malignant transformation (2, 10).

The case reported in this article aligns broadly with the characteristics described above. The patient underwent small bowel resection for lymphoma in 2020, followed by chemoradiotherapy. Upon admission, he had low albumin levels and poor overall nutritional status, meeting basic conditions associated with anal fistula carcinogenesis. Additionally, prior abdominal surgery may contribute to carcinogenesis. A previous study analyzing 61 cases of Crohn’s-related anal fistula carcinogenesis found that 64% of patients had a history of abdominal surgery, with procedures ranging from small bowel resection to total proctocolectomy (3). This case supports that association. Although the patient had a long-standing history of anal fistula, the condition was not persistently active over the past decade. He reported no recurrence for more than 10 years following the initial surgery, and anal discomfort only began 1 year ago, which differs from most of the previously reported cases. Unfortunately, HPV testing was not performed during hospitalization; however, the patient denied any history of HPV infection, blood transfusion, or unprotected sexual intercourse.

Diagnosing anal fistula-related malignancies is often challenging. In this case, routine endorectal ultrasound at admission suggested that the lesion had penetrated the submucosa, prompting a recommendation for pathological evaluation to confirm the diagnosis. Subsequently, enhanced MRI was performed, but no specific abnormalities were observed. Lad et al. compared MRI findings from six cases of Crohn’s disease-related anal canal cancer (four mucoid adenocarcinomas and two squamous cell carcinomas) with 18 cases of Crohn’s disease-related anal fistulas. They found that patients with anal canal cancer showed irregular inner wall contours and delayed mild enhancement of internal tissues on MRI (11). In the present case, enhanced MRI did not reveal these features, possibly because the malignancy was still in an early stage and lacked typical imaging characteristics. Endorectal ultrasound demonstrated notable advantages in this diagnostic process. Its ability to provide real-time, dynamic observation allows for multi-directional assessment of the lesion’s relationship to surrounding tissues by adjusting the probe angle and pressure. This technique can detect subtle infiltrative changes, thus offering more detailed information for clinical evaluation. However, its limitations must also be considered. Long-standing chronic inflammation in anal fistulas can cause local edema and fibrosis, increasing the risk of mistaking inflammatory adhesions for tumor infiltration. As such, relying on a single imaging modality is insufficient. In clinical practice, combining multiple diagnostic tools, such as ultrasound and MRI, can enhance diagnostic accuracy. Ultimately, however, the final diagnosis must depend on the gold standard of pathological confirmation.

Given the rarity of these diseases, no standardized treatment protocol currently exists. Most available information on the management of anal fistula-related malignancies is derived from case reports (1). A retrospective study by Kotsafti et al. involving 36 patients with anal fistula-related squamous cell carcinoma reported that 23 patients received treatment based on the Nigro protocol. Of these, 8 underwent abdominoperineal resection (APR) due to residual disease following chemoradiotherapy. Prognostic data were unavailable for 11 of the 23 patients. Among those treated with the Nigro protocol combined with APR, 4 were lost to follow-up, 2 died within 2 years, and 2 remained alive during the follow-up period (3–5 years) (2). For anal fistula-related adenocarcinoma, APR remains the primary treatment. In advanced cases, neoadjuvant therapy and chemoradiotherapy can improve patient quality of life (12).

## Conclusions

This case report presents a patient with anal fistula-related squamous cell carcinoma. Although the patient had a history of anal fistula spanning more than 10 years, the condition did not recur continuously. After healing from the initial surgery, he experienced an asymptomatic period lasting over a decade, with anal discomfort only emerging one year ago. Notably, postoperative pathology from the surgery one year prior revealed no abnormalities. This pattern of disease progression differs markedly from previously reported cases of anal fistula carcinogenesis and offers a novel perspective on the clinical characteristics of this rare condition.

## Data Availability

The original contributions presented in the study are included in the article/supplementary material, further inquiries can be directed to the corresponding author/s.
